# Amyotrophic lateral sclerosis: a dying motor unit?

**DOI:** 10.3389/fnagi.2013.00007

**Published:** 2013-03-26

**Authors:** Maria Piotrkiewicz, Irena Hausmanowa-Petrusewicz

**Affiliations:** ^1^Department of Engineering of Nervous and Muscular System, Nałęcz Institute of Biocybernetics and Biomedical Engineering, Polish Academy of SciencesWarsaw, Poland; ^2^Neuromuscular Unit, Medical Research Center, Polish Academy of SciencesWarsaw, Poland

## Introduction

ALS is a late-onset neurodegenerative disease of unknown etiology. Since it was first described (Charcot, 1874), several mechanisms, such as oxidative stress, glutamate excitotoxicity, glial cell pathology, or aberrant RNA metabolism (for review, see Vucic and Kiernan, 2009). Recently the evidence accumulates that mitochondrial damage and defective axonal transport may play a pivotal role (Shi et al., 2010; Cozzolino and Carri, 2012). Nowadays, a multifactorial origin is widely accepted for the neurodegeneration in ALS, but the triggering mechanism(s) underlying its initiation remain to be defined.

One of the issues, which are still unresolved, is the triggering site. ALS is classically thought to be a disease that causes the progressive loss of upper and lower motoneurones (MNs) followed by axonal degeneration and muscle atrophy, resulting in the virtually complete disappearance of spinal and cortical MNs (Charcot, 1874; Strong and Rosenfeld, 2003). This point of view is still prevalent among clinical neurologists, and ongoing debates focus mostly on the question of which MN subsystem (upper or lower) is affected first (Chou and Norris, 1993; Eisen et al., 1996; Eisen, 2009; Van Der Graaff et al., 2009).

For obvious reasons, it is not possible to directly characterize the properties of MNs in ALS patients. Therefore, a significant proportion of the research on ALS takes place in animal models of the disease that carry mutations in the Cu/Zn superoxide dismutase gene (the SOD1 mice and rats). Although these mutations are responsible only for about 2.5% of all the ALS cases, the pathology in SOD1 animals largely resembles clinical features of human sporadic ALS.

In recent years, research in SOD1 animal ALS models has found MN pathology to begin at the distal axon terminals and to proceed in a “dying back” pattern (Fischer et al., 2004; Xie et al., 2005; Parkhouse et al., 2008; Shi et al., 2009; Carrasco et al., 2010). Below, we will present a piece of experimental evidence that, in our opinion, may be interpreted in favor of this last view.

## The single motor unit case

The detailed information on the methods applied and the patients investigated is given in the study published earlier (Piotrkiewicz et al., 2008). Briefly, in the study participated 20 patients, aged 31–75 (mean 55.0 years) and diagnosed as having definite ALS according to El Escorial criteria (Brooks, 1994). Motor unit potentials (MUPs) were picked up from the brachial biceps by intramuscular disposable wire electrodes, amplified and transferred for off-line analysis to a PC computer by an A/D converter with sampling rates 10–20 kHz.

**Figure 1:**
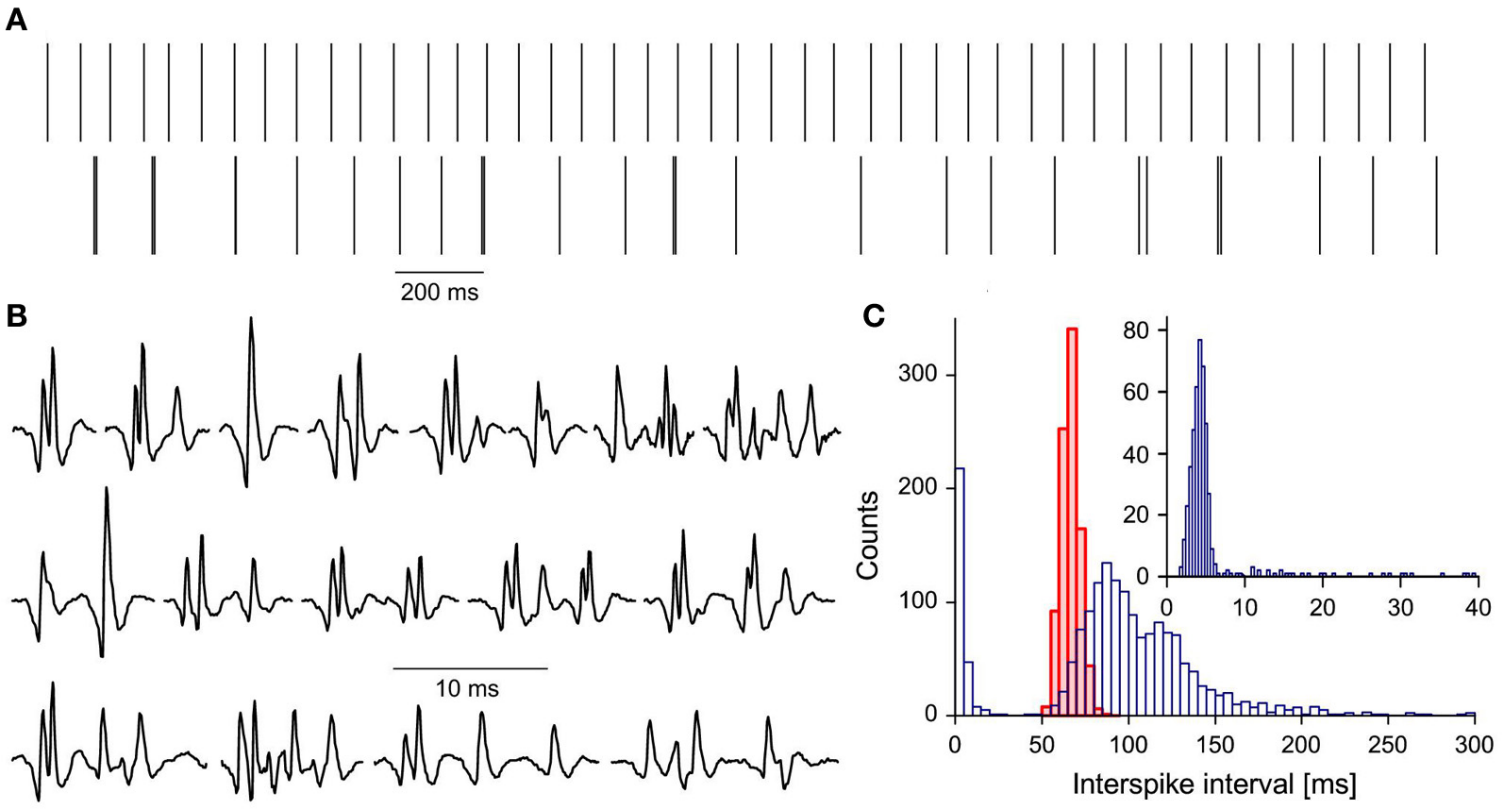
**Characteristics of E-MU: (A) discharge patterns: upper trace, N-MU; lower trace, E-MU.** Each discharge represented by a vertical line; **(B)** a variety of E-MU potential shapes; **(C)** histograms of interspike intervals: N-MU, thick red line, shaded area, E-MU, thin blue line, open area; insert: histogram of doublet and “outsider” intervals of E-MU in expanded scale. Bin width: 5 ms for pooled histogram, 0.4 ms for inset.

Among 124 single motor units (MUs) analyzed in this study we found one (E-MU), which strikingly differed from all others. It was recorded from the brachial biceps of 49 years old patient with 50% force deficit (as compared with average maximum force of control subjects). The firing pattern of the E-MU was very irregular, which could not be ascribed to the fluctuating synaptic inflow, since the other MU (N-MU) recorded simultaneously discharged very regularly (Figure 1A).

E-MU exhibited also an exceptional variability of potential shape due to the high jitter and changes in the number of individual components (Figure 1B). It was often difficult to decide, whether the given component was an integral part of MU potential that was sometimes blocked, a satellite, a doublet of the motoneuronal origin, or ectopic discharge. Because of these ambiguities E-MU was excluded from the analysis of doublet firing. Figure 1C presents the interspike interval histogram of E-MU, compared with that of N-MU. The initial peak of this histogram (0–20 ms), which includes the short intervals between potential pairs recognized as doublets, is presented with expanded time scale and shorter bin width in the insert to Figure 1C. The following maximum (at 85 ms) corresponds to the regular discharges and the next one (at 120 ms) is due to the prolonged post-doublet intervals, typical feature of double discharges. The histogram reveals also several intervals, so-called “outsiders,” which duration is longer than officially accepted upper limit of doublet intervals (20 ms, according to AAEM, 2001), but shorter than the lower limit of the regular interval histogram. In our previous study (Piotrkiewicz et al., 2008) such pairs of potentials were classified also as doublets, since their intervals were visibly shorter and always followed by post-doublet interval significantly longer as compared with the regular discharge. The E-MU discharge was characterized by exceptionally high percentage of “outsiders,” as compared with other doubling MUs from ALS patients.

## Possible explanation

De Carvalho (2000) described two distinct types of fasciculation potentials recorded from ALS patients. Fasciculation potentials of the first type, which were usually recorded from strong muscles, were stable, simple and the same potentials could be recruited both voluntarily and by transcranial magnetic stimulation. Fasciculation potentials of the second type were complex, unstable, tended to have lower discharge rates and were generated only spontaneously. It was suggested that the fasciculations of the first type arise at the spinal (or upper) MN level and those of the second type, in distal axonal sprouts. This suggestion was further confirmed by a recent study of Kleine et al. (2008).

In SOD1 mice (animal model of ALS). Schaefer et al. (2005) found two populations of MUs: one (1) with well-preserved axonal branches, sometimes enlarged as the result of reinnervation, and the other (2) with degenerating axon terminals, sometimes with no neuromuscular contacts at all. The latter finding is in line with growing number of studies in SOD1 mice suggesting that the disease may start in neuromuscular junction and proceed with the “dying-back” pattern through an axon to the MN (e.g., Fischer et al., 2004).

If also in the human sporadic ALS the disease began in motor endplates, then one might expect the presence of two populations of MUs: one which is still intact and the other being under process of losing their neuromuscular contacts and thus on the way to degeneration. The first one would correspond to the population (1) in Schaefer's study and would produce fasciculations of de Carvalho's first type as well as doublets, both generated by hyperexcitable MNs. The second one would correspond to the Schaefer's population (2); the affected, hyperexcitable axonal collaterals (Bostock et al., 1995) could generate ectopic activity (fasciculations of de Carvalho's second type). Our MU with unstable potential shape, irregular low-rate discharge, big percentage of “outsiders,” but still under voluntary control, might represent the intermediate type: a MU, which is loosing contacts with its muscle fibers. It is conceivable that the time from the first noticeable symptoms to the complete loss of MN control upon its muscle unit may be short and thus the majority of MUs, which can be voluntarily driven, would belong to the yet unaffected population. This may be the reason why among 124 analyzed ALS MUs we found only one with the characteristics described above.

## Concluding remarks

Could it be possible that human ALS also begins in motor endplates and proceeds via axons in a “dying-back” pattern?

In fact, abnormalities in motor axon properties are often reported in ALS patients (Bostock et al., 1995; Mogyoros et al., 1998; Stephanova et al., 2001; Priori et al., 2002; Koszewicz et al., 2005; Kanai et al., 2006; Vucic and Kiernan, 2006). The pathology of motor axons has been shown to be more pronounced distally (Nakata et al., 2006; Noto et al., 2011). Certain lines of evidence derived from clinical neurophysiology of ALS (Eisen and Swash, 2001) could be also interpreted as favoring the “dying back” hypothesis of MN degeneration. For example, the marked fatigue observed early in the disease could indicate an endplate dysfunction. The routine test to diagnose the impairment of neuromuscular transmission in Myasthenia gravis, typical motor endplate disease, is the investigation of change in MU potential amplitude due to repetitive nerve stimulation. The amplitude decrements in such a test indicate the impairment of neuromuscular transmission. Decremental responses to stimulation were observed also in ALS (Bernstein and Antel, 1981; Killian et al., 1994; Wang et al., 2001). Iwanami and colleagues (2011) found even the incidence of positive decrements higher in the ALS than in the Myasthenia gravis patients.

The ongoing debates between clinical neurologists focus essentially on the question of which MN subsystem is affected first. The term “dying back” was used in this debate to characterize the hypothesis that the ALS is primarily a lower MN disease spreading to upper MNs (Van Der Graaff et al., 2009). However, if ALS pathology indeed began in axons, then the lower and upper MNs could degenerate independently of each other. This possibility was recently suggested by several authors (Terao et al., 1999; Attarian et al., 2008; Agosta et al., 2009).

Recently, the question of possible heterogeneity of ALS is often raised (Adamek et al., 2002; Beghi et al., 2007; Bastos et al., 2011; Chiò et al., 2011; Van Den Berg, 2011). It is thus possible that different ALS phenotypes may be initiated at different sites. In any case, the proper defining of disease initiation site would be an important step toward eventual discovery of the factor triggering the disease and the target of its possible treatment.

In this opinion we concentrated on the problem of ALS initiation site, favoring one of several possibilities. We are aware that there could be an alternative explanation of firing patterns observed in our E-MU. If, however, this paper evokes a discussion on the possible initiation site of ALS, this may be a little step toward final unraveling of ALS etiology.
